# Inducible displacement CT for implant loosening detection: a scoping review on methods, validation, and challenges

**DOI:** 10.2340/17453674.2026.45512

**Published:** 2026-02-23

**Authors:** Maaike A TER WEE, Johannes G G DOBBE, Arthur J KIEVIT, Matthias U SCHAFROTH, Mario MAAS, Leendert BLANKEVOORT, Geert J STREEKSTRA

**Affiliations:** 1 Department of Orthopaedic Surgery and Sport Medicine, Amsterdam UMC, location AMC, Amsterdam; 2 Department of Biomedical Engineering and Physics, Amsterdam UMC, location University of Amsterdam, Amsterdam; 3 Amsterdam Movement Sciences, Musculoskeletal Health, Amsterdam; 4 Department of Radiology, Amsterdam UMC, location University of Amsterdam, Amsterdam, The Netherlands

## Abstract

**Background and purpose:**

Inducible displacement CT (ID-CT) is an emerging method for diagnosing implant loosening by (i) acquiring CT scans under different joint loading conditions, (ii) analyzing scans via segmentation and registration, and (iii) quantifying and visualizing relative implant–bone displacement. With multiple centers approaching these steps differently, this scoping review aimed to summarize current methodologies and key challenges.

**Methods:**

PubMed, Cochrane, and Embase were searched for clinical and experimental ID-CT studies on spinal and arthroplasty implants. Data was extracted using a table based on updated CT-radiostereometric analysis (RSA) guidelines, including study characteristics, CT acquisition parameters, image analysis methods, validation approaches, outcomes, and loading protocols. Diagnostic studies were assessed with QUADAS-2.

**Results:**

22 studies were included concerning the hip (10), knee (7), spine (4), and wrist (1), covering clinical and experimental (phantom and cadaveric) designs. Loading protocols varied widely, applying compressive, rotational, and angular forces with external stabilization or loading devices. CT acquisition ranged from µCT to conventional and weight-bearing CT, with variation in kVp, mAs, reconstruction spacing, and metal artefact reduction. Image-analysis workflows were broadly similar, though reporting of algorithms and displacement metrics was often incomplete. Diagnostic validation was limited by variable reference standards, non-prespecified loosening thresholds, and non-blinded assessments, undermining true performance. Technical validation, although often precise, did not cover the full ID-CT pipeline (i.e., complete loading protocol), leaving key sources of measurement variance untested.

**Conclusion:**

We showed that ID-CT is used with a wide variation in approach and limited reporting prevents the establishment of its true diagnostic accuracy.

Early implant migration can be detected with radiostereometric analysis (RSA) and is a predictor of later aseptic loosening [1-7]. RSA is accurate in upper and lower extremities [7,8], but clinical application is limited by invasive marker implantation, biplanar X-ray calibration cages, and long-term follow-up to detect migration. To address these limitations, CT-based RSA (CT-RSA) was introduced in the early 2000s [9], demonstrating comparable accuracy [10]. In parallel, inducible displacement RSA showed that implant displacement under induced load relates to long-term migration and can predict failure [11-14]. Inducible displacement CT (ID-CT) builds on this by using CT imaging under different loading (provocation) conditions to directly measure implant displacement, enabling immediate stability assessment and avoiding many (CT-)RSA limitations, such as marker implantation, calibration cages, and long-term follow-up. It shows promise as a diagnostic tool for loosening but presents challenges, including the need for controlled, reproducible loading, managing metal artefacts, standardized analysis, and defining clinically meaningful thresholds for loosening.

Migration-based CT-RSA and load-based ID-CT share a methodological foundation, with similar image analysis workflows and validation protocols. Evidence from CT-RSA thus contributes to ID-CT’s conceptual development, and a recent CT-RSA review [10] provides a key reference. However, that review focused on migration and did not address challenges specific to ID-CT. Our scoping review therefore addresses the following questions: what methodological approaches are used to assess implant loosening with ID-CT, and what variability is reported across studies? This review summarizes literature covering loading protocols, CT settings, image analysis, and validation approaches, and discusses key challenges in the broader CT-RSA context. 

## Methods

This scoping review was conducted in accordance with the PRISMA-ScR checklist [15] (see Supplementary data).

### Search strategy

Due to inconsistency in terminology, making it unclear whether implant displacement reflects migration-over-time or load-induced, a broad search strategy was used. The primary search in PubMed used MeSH and free-text terms: “arthroplasty[MeSH] AND (migration OR motion OR displacement) AND CT.” As a secondary step, the PubMed, Embase, and Cochrane search from Van de Vusse et al. [10] was replicated, which used “arthroplasty, AND migration AND computed tomography” as main terms. Reference lists of included studies and relevant reviews were screened manually. The search covered English-language, peer-reviewed articles published from January 2000 to March 2025. References were managed in Endnote X9 (Clarivate Analytics, Philadelphia, PA, USA).

### Eligibility criteria

Experimental and clinical studies were included if they investigated inducible displacement using CT to assess the stability of joint or spinal implants and reported either quantitative or qualitative outcomes. Exclusion criteria included use of conventional RSA without CT, studies focused solely on implant position at a single time point, absence of implants (e.g., only healthy controls), and reports or conference abstracts.

### Study selection, data extraction, and synthesis

Study selection and data extraction were performed by 1 reviewer (MAtW). To ensure no studies were overlooked, both migration-over-time and load-induced displacement studies were considered during abstract screening, with the final selection at the full-text phase limited to ID-CT studies. A standardized table was developed for this review, extracting data items according to the updated (CT-)RSA guidelines [8], including: study characteristics (design, population, sample size), technical specifications (CT acquisition and reconstruction settings), image analysis approaches (software, displacement metrics, coordinate systems), reliability and validation measures (accuracy, precision, reference standards), outcomes (displacement and performance metrics), along with loading methodology (loading protocols and devices). This scoping review was not prospectively registered, as registration is not required by PRISMA-ScR guidelines.

### Methodological quality

Risk of bias and applicability for diagnostic ID-CT studies were evaluated with the QUADAS-2 (Quality Assessment of Diagnostic Accuracy Studies-2) tool [16] by 1 reviewer (MAtW). Potential bias was identified across the 4 domains: patient selection, index text, reference standard, and flow & timing, when signaling questions received negative responses.

### Terminology

Terminology for CT-based implant displacement analysis varies across studies, with many terms failing to distinguish migration-over-time from load-induced displacement. We find “CT-RSA” ambiguous, as CT involves no stereo acquisition. In this review, we adopt inducible displacement CT (ID-CT), in line with recent guidelines [8], and reserve CT migration-analysis for displacements measured over time.

### Funding, use of AI, and disclosures

The authors did not receive funding for this study. Microsoft Copilot was used to assist with text editing and language refinement during manuscript preparation. The authors reviewed and approved all content. No content, data interpretation, or original text was generated by the tool.

Complete disclosure of interest forms according to ICMJE are available on the article page, doi: 10.2340/17453674.2026.45512

## Results

### Search

The initial search yielded 1,798 records (933 from the primary search and 865 from Van de Vusse et al. [10]) (Figure). After removing 345 duplicates, 1,453 unique studies remained for title and abstract screening. Based on inclusion criteria, 77 were selected for full-text evaluation, of which 26 were excluded. An additional 8 studies were identified through reference screening, resulting in 59 studies. Full-text evaluation identified 37 studies on CT migration analysis and 22 studies on ID-CT. All characteristics of the ID-CT studies are summarized in Table S1 (see Supplementary data). The studies were grouped by anatomy: hip (10), knee (7), spine (4), and wrist (1).

**Figure:**
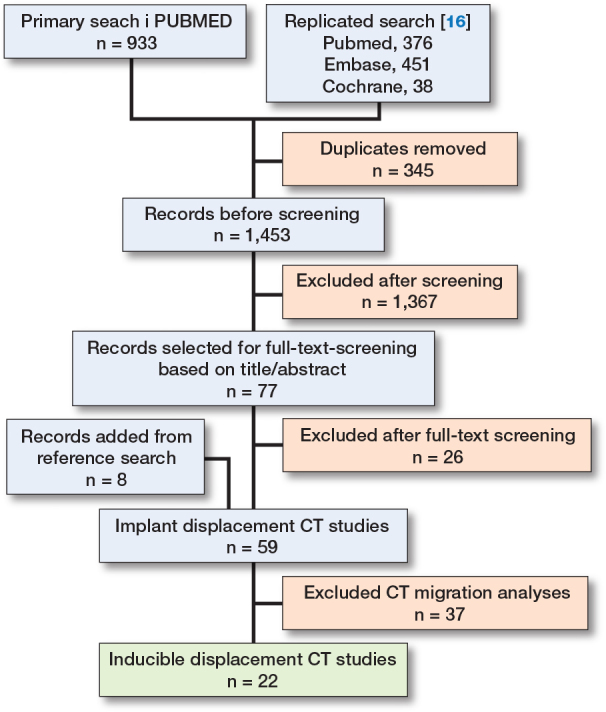
Flowchart of inclusion of records in the current scoping review.

### Loading protocols

#### Hip

10 studies investigated total hip arthroplasty (THA), focusing on the acetabular cup [17], femoral stem [18-23], or both components [24-26]. 5 of these applied manual internal and external leg rotation to the limit of motion, with external stabilization using sandbags or foam pads [24-26], or without specified stabilization [17,18]. 5 cadaveric studies used compressive axial loading (stepwise max 1,400 N [20], 1,800 N [22], or 2,000 N [21]) or both compression and torsional loading (1,800 N and 17 Nm [19] or bodyweight based [23]) using custom loading devices in a µCT setup.

#### Knee

7 studies examined total knee arthroplasty (TKA), focusing on tibial [27-30] or both tibial and femoral components [31-33]. 4 studies applied varus–valgus loading using 4-point bending devices set to apply standardized 20 Nm moment to the knee [27-30]. 1 study used compressive loading by comparing seating and standing in a weight-bearing CT [32]. 1 study combined both torsional (internal–external rotation) and angular (varus–valgus) loading using external stabilization (foam pads, lashing straps) [31], and 1 study on megaprostheses used torsional loading only [33].

#### Spine

4 studies investigated disc replacements, 2 cervical [34,35] and 2 lumbar [36,37]. Cervical studies used voluntary flexion and extension with patients lying on their side with pillow support [34,35]; 1 included phantom model validation using cadaveric vertebrae [34]. 1 lumbar study used the same phantom model setup to validate the method [37], while the other patient study used provoked flexion–extension with a custom jig and blocks to reach Visual Analogue Scale (VAS) pain scores of 8/10 or maximum range within the CT gantry [36].

#### Wrist

1 study examined wrist arthroplasty using custom orthoses to achieve maximum flexion and extension [38].

### CT settings and dose

12 of 22 studies reported CT parameters, including kVp, mAs, slice thickness, and pixel or voxel spacing. kVp ranged from 90–140, mAs from 20 (knee weightbearing CT [32]) to 180 (hip [25]). Slice thickness was reported inconsistently; many studies listed only increments or spacing [26,34,36,37], which does not reflect true reconstruction thickness. In patient studies, reported slice thickness or voxel spacing ranged from 1.25 mm (earlier study [17]) to 0.45 mm (recent study [30]). In experimental µCT setup this was 35–36 μm [19-23]. Effective dose (ED) was reported in 8 studies, ranging from 0.01 mSv in the wrist [38] to 4.50 mSv in the hip [25]. Hip scans had the highest radiation doses (1.5–4.5 mSv), followed by knee (0.04–0.5 mSv [32] and 1.2 mSv [29]), lumbar spine (0.68 mSv) [36], cervical spine (0.33 mSv) [34,35], and wrist (0.01 mSv) [33]. Reported doses are for single scans, while all inducible displacement exams required 2 scans.

### Image analysis methodology and software

All software implemented a 3-step approach: segmentation (threshold-based region growing, manual delineation, or automatic detection of radiopaque beads), registration (landmark-, surface-, or intensity-based), and displacement calculation with visualization (heatmaps). Roughly, 5 techniques can be distinguished (Table 1). The underlying algorithms of the commercial IMA software are, to our knowledge, not available in the literature, limiting understanding of its methods.

**Table 1:** Overview of image analysis methodology and software used in ID-CT studies

Software/tool	Origin/developer	Joint(s)	Segmentation method	Registration method	Displacement calculation/metrics	Notes
IMA	Sectra, Linköping, Sweden. Methods developed by Olivecrona 2002 [9] and Jedenmalm 2008 [69]	Hip [24-26] Kne [31,33], Wrist [38]	Unknown **^a^**	Unknown	Visual assessment (heatmaps); displacement in mm (specific metrics not disclosed)	Commercially available. Evolved from 3D Volume fusion tool
3D volume fusion	Noz 2001 [70], Olivecrona 2002 [9], and Gorniak 2003 [71]	Hip [17], Spine [34-37]	Unknown **^a^**	Rigid-body transformation based on anatomical landmarks	Cross-correlation on axial slices before and after transformation	
AtMoves	Amsterdam UMC, the Netherlands	Knee [27-30]	Threshold-connected region growing; marching cubes for polygon mesh extraction [72]	Grey-value correlation between double-contour models (cortical vs soft tissue) optimized via Nelder–Mead simplex [73]	x, y, z translations/rotations, total translation, total rotation, mTRE, MTPM	Local CS at tibial plateau center; defined by eigenvector analysis
V3MA	RSAcore, Leiden, the Netherlands	Hip [18], Knee [32]	3D Slicer (v5.2.2); segmentation mask defines sampled region	Elastix toolbox [74]; normalized cross-correlation; adaptive stochastic gradient descent with multi-resolution [42]	Translations, rotations, and MTPM (same definitions as AtMoves)	Coordinate system aligned with standard RSA; origin at implant center of mass
µCT-analysis	Self-developed	Hip [19-23]	Bead centers segmented using Amira (v6.0.1, FEI company) or custom software	Generally unspecified; 1 study [19] used (ICP with rigid-body transformations and median absolute deviation filtering [75]	Translation along normal (x, y) and tangential (z) axes of femoral stem surface	Used tantalum or stainless-steel beads

CS: coordinate system, FEI: Field Electron and Ion Company (now Thermo Fisher Scientific). ICP: iterative closest point, ID-CT: inducible displacement computed tomography, IMA: Implant Movement Analysis, MTPM: maximum total point motion. mTRE: mean target registration error, RSA: radiostereometric analysis, UMC: University Medical Centre, V3MA: Volumetric Matching Micromotion Analysis

a The current studies do not specify segmentation and registration methods, but refer to previous studies which themselves provide minimal methodological detail.

### Diagnostic and technical validity

Validation approaches were broadly divided into (i) diagnostic, comparing ID-CT for distinguishing loose versus fixed implants against an accepted measurement method, i.e., a reference standard (Table 2), or (ii) technical, in which the measurement accuracy, precision (i.e., repeatability), and robustness of ID-CT were assessed in clinical and experimental studies (Table 3). To clarify which image analysis methodology was used in each validation study, this is specified in the text and in Tables 2 and 3 by indicating one of the 5 techniques: IMA, 3D Volume fusion, V3MA, AtMoves, or µCT analysis (see Table 1).

**Table 2:** Overview of studies evaluating the diagnostic performance of ID-CT, i.e., comparing ID-CT for distinguishing loose vs fixed implants against a (combination of) reference standard(s). “Patient-level” refers to analyses where a case was classified as “loose” if 1 or both components were loose, “component-level” refers to analyses where each component was classified separately

Joint First author, year, ref.	Technique	Component(s)	Reference standard(s)	Findings	ID-CT findings	Patient selection
Hip						
Sandberg 2021 [24]	IMA	Cup, stem, tibial comp	Intraoperative	Cups: 5 loose, 4 fixed Stems: 6 loose, 4 fixed Tibial comp: 1 fixed	Cups: 5 loose, 2 fixed, 2 inconclusive Stems: 4 loose, 5 fixed, 1 inconclusive Tibial comp: 1 fixed	Inconclusive radiography, suspected implant loosening
Sandberg 2022 [25]	IMA	Cup, stem	Intraoperative Radiography PROMs	17 loose, 40 fixed (patient-level). Cups: 11 loose, 46 fixed Stems: 6 loose, 51 fixed	Sensitivity: 77%, specificity 100% (patient-level) Cups: 7 loose, 50 fixed Stems: 6 loose, 51 fixed	Inconclusive radiography Positive ID-CT indicated revision surgery
Listopadzki 2025 [26]	IMA	Cup, stem	Intraoperative PROMs	8 loose, 72 fixed (patient-level)	Sensitivity: 100%, specificity 100% 8 loose, 72 fixed (patient-level) Intraoperative only (n = 23): 7 loose, 16 fixed	Unknown
Polus 2025 [18]	V3MA	Stem	(ID-)RSA	MB-RSA (5-year follow-up) shows well-fixed (< 0.2 mm/year) for all patients	No difference in ID in x, z translation or x, y, z rotation between RSA and CT Difference in y translation, TT and TR	Previously participation in 2-year prospective RSA study
Knee						
Wretenberg 2021 [31]	IMA	Tibial comp, femoral comp	Intraoperative	8 loose, 0 fixed (patient-level)	8 loose, 0 fixed (patient-level)	Suspected loosening, inconclusive radiography. Positive ID-CT indicated revision surgery
Buijs & Kievit 2025 [29]	AtMoves	Tibial comp	Intraoperative	24 loose, 10 fixed (component-level)	Sensitivity: 91% (CI 72–97), Specificity: 72% (CI 43–90), PPV: 87% (CI 68–95), NPV: 80% (CI 49–95) Optimized loosening threshold **^a^** (component-level)	Scheduled for revision surgery based on local protocol
Hext 2025 [32]	V3MA	Tibial comp, femoral comp	(ID-)RSA	MB-RSA (5-year follow-up, component-level). Tibial comp: 1/17 continuous migration (> 0.2 mm/year) Femoral comp: 3/17 continuous migration	Tibial comp: No difference in ID in x, y, z translation or y, z rotation between RSA and CT. Difference in x rotation, TT, TR, and MTPM. Femoral comp: No difference in ID in x translation, x, y, z rotation and MPTM between RSA and CT Difference in y and z translation and TR	Previous participation in 5-year prospective RSA study
Svensson 2025 [33]	IMA	Tibial comp, femoral comp	Intraoperative	11 loose, 2 fixed (3 did not undergo revision surgery)	Sensitivity 100%, specificity 75%, PPV: 90%, NPV: 100%. 9 loose, 3 fixed (patient-level)	Suspected loosening, inconclusive radiological findings. Positive ID-CT indicated revision surgery
Spine						
Skeppholm 2015 [35]	3D Volume fusion	Upper and lower comp	Intraoperative	Upper comp: 3 loose, 0 fixed. Lower comp: 0 loose, 3 fixed.	Upper comp: 3 loose, 0 fixed. Lower comp: 0 loose, 3 fixed	Positive ID-CT indicated revision surgery

CI: 95% confidence interval. CT: computed tomography. Comp: component. ID: inducible displacement. MB-RSA: model-based RSA. MTPM: maximum total point motion. PROMs: patient-reported outcome measures. RSA: radiostereometric analysis. TR: total rotation (square root of the quadratic sum of x, y, z rotations). TT: total translation (square root of the quadratic sum of x, y, z translations).

a “Optimized loosening threshold” refers to a cutoff displacement value, determined retrospectively against the reference standard, that best distinguishes fixed from loose implants, achieving maximum sensitivity and specificity.

**Table 3:** Overview of technical validation ID-CT studies, i.e., evaluating measurement accuracy, precision (i.e., repeatability), and robustness. Variance source(s) represent the origins of measurement variability within the ID-CT workflow that are addressed in the respective study design. The Experiment column describes the study setup: *zero-displacement examinations* (apparent displacement from repeated scans in unloaded conditions), *double-examinations* (apparent displacement from repeated scans under the same loading condition), *pilot* (no refence standard, exploratory application in subjects), or other setups

Joint Study	Technique	Subject	Variance source(s)’	Experiment	Major findings
Hip					
Olivecrona 2008 [17]	3D Volume fusion	Patient, cup	Subject variability Image analysis	*Double image analysis* (2 observers); visually graded (< 1 mm, 1 mm error, or > 1 mm registration error) and numerically (distance difference of landmarks)	No difference in ID-CT diagnosis between observers. Visual and numerical (landmark) registration error < 1mm
Gortchacow 2011 [20]	µCT-analysis	Cadaver, femoral stem	Scanning Image analysis	1.96*SD of relative displacement between stem beads at all loading conditions 1.96*SD of relative displacement between bone beads in unloaded condition (*zero-displacement examination*) Displacement between actual and reference position of the beads (*pilot*)	Stem beads maximal error: x-, y-axis 5.2 μm, z-axis 9.4 μm No load dependency on the x, y and z-axis Bone beads error: 9 μm Displacement z-axis increased with load, up to 60 μm at 1,400 N
Gortchacow 2012 [21]	µCT-analysis	Cadaver, femoral stem	Subject variability Scanning Image analysis	1.96*max(SDx, SDy, SDz) of relative displacement between bone beads in unloaded conditions (3rd & 4th scans) with random repositioning in the scanner (*zero-displacement examination*)	Bone beads maximal error: 15 μm in x, y, z directions
Malfroy Camine 2015 [22]	µCT-analysis	Cadaver, femoral stem	Scanning Image analysis	Absolute displacement of bone markers from unloaded (1st) to unloaded (2nd) scan (*zero-displacement examination*) Displacements of bone beads from (4th) unloaded to (3rd) loaded scan (*pilot*)	Bone beads absolute displacement error: 20 μm for 90% of beads. Displacement amplitude 5.5–50.7 μm (mainly vertical), mean 25.9 μm across stem surface
Malfroy Camine 2016 [19]	µCT-analysis	Cadaver, femoral stem	Loading Scanning Image analysis	1.96*SD of relative displacement between implant and bone beads of 3 pairs of successive unloaded scans, i.e., bias (*zero-displacement examination*) SD of relative displacement between implant and bone of 3 pairs of repeated loaded (compression and torsion) scans, i.e., repeatability. Repeatability limit = 1.96*√2*sr Displacements across stem surface between unloaded and loaded scan (*pilot*)	Bias: max 5.1 μm Repeatability: SD range 3.1–4.0 μm Repeatability limit: compression 10.6 μm, torsion 11.5 μm Peak displacement range: 24 μm (compression), 49 μm (torsion)
Malfroy Camine 2018 [23]	µCT-analysis	Cadaver, femoral stem	Subject variability	Displacements across 12 Gruen zones (1–3, 5–10, 12–14) between loaded (2nd) and unloaded (3rd) scan (*pilot*)	Compression: mean absolute displacement 19.5 (SD 5) μm (collarless), and 43.3 (SD 33.1) μm (collared) Torsion: mean absolute displacement 96.9 (SD 59.8) μm (collarless), and 118.7 (SD 45.0) μm (collared)
Polus 2025 [18]	V3MA	Patient, femoral stem	Subject variability Scanning Image analysis	*Double examinations* in external rotation Mean relative displacement implant and bone Bias = mean of double examinations, precision = 1.96*SD	Bias: TT 0.055 mm, TR 0.102° Precision: TT 0.100 mm, TR 0.167°
Knee					
Kievit & Buijs 2023 [27]	AtMoves	Cadaver, tibial comp	Subject variability Scanning Image analysis	Mean (SD) of relative displacement between implant and bone of repeated (10x) unloaded scans (*zero-displacement examination*) Differentiation between known fixation states (loose vs. fixed)	mTRE 0.07 (SD 0.03) mm; screw-axis rotation 0.13° (SD 0.04); MTPM 0.12 (SD 0.03) mm Significant difference (P < 0.001) between loose (mTRE 1.06 [SD 0.33] mm; screw-axis rotation 2.44° [SD 0.97]; MTPM 2.18 [SD 0.86] mm) and fixed (mTRE 0.60 [SD 0.21] mm; screw-axis rotation 0.67° [SD 0.66]; MTPM 0.31 [SD 0.78] mm) for all metrics
ter Wee 2023 [28]	AtMoves	Cadaver tibial comp	Subject variability Scanning Image analysis	Mean (SD) of relative displacement between implant and bone of repeated (10x) unloaded scans using 2 image analysis protocols (*zero-displacement examination*) Differentiation between known fixation states (loose vs. fixed) using 2 image-analysis protocols	100% tibia protocol: mTRE 0.05 (SD 0.02) mm; screw-axis rotation 0.08° (SD 0.04); MTPM 0.07 (SD 0.04) mm 20% tibia protocol: mTRE 0.08 (SD 0.06) mm; screw-axis rotation 0.08° (SD 0.03); MTPM 0.11 (SD 0.06) mm Improved differentiation between loose and fixed implants for 20% vs 100% tibia protocol (mTRE and MTPM)
Buijs & Kievit 2025 [29]	AtMoves	Patient tibial comp	Subject variability Image analysis	*Double image analysis:* intra- and interrater (3 raters)	Intrarater reliability (ICC): 0.74–0.96 Interrater reliability (ICC): 0.89–0.98
Buijs & Ter Wee 2025 [30]	AtMoves	Patient tibial comp	Subject variability Loading Scanning Image analysis	Repeated loaded scans (varus and valgus) by 2 operators 2 image analysis protocols	100% tibia protocol: ICC 0.64–0.84, SEM: mTRE 0.10 mm, screw-axis rotation 0.16°, MTPM 0.15 mm 20% tibia protocol: ICC 0.17–0.31, SEM: mTRE 0.06 mm, screw-axis rotation 0.14°, MTPM 0.10 mm
Hext 2025 [32]	V3MA	Patient femoral and tibial comp	Subject variability Scanning Image analysis	*Double examinations* in seated position Precision = 1.96*SD	Tibial comp precision: TT 0.056 mm, TR 0.118°, MTPM 0.141 mm Femoral comp precision: TT 0.071 mm, TR 0.128°, MTPM 0.117 mm
Spine					
Svedmark 2008 [37]	3D Volume fusion	Phantom lumbar disc	Scanning Image analysis	Imposed (known) displacements Accuracy limit = 1.96*average difference Repeatability measurements Repeatability = 1.96*SD	Accuracy: 3D translation 0.56 mm, sagittal 0.45 mm, coronal 0.46 mm, axial 0.45 mm Repeatability 0.35 mm
Svedmark 2011 [34]	3D Volume fusion	Patient and phantom cervical disc	Scanning Image analysis	Patient: no reference (*pilot*) Model: imposed (known) displacements (accuracy [76]) *Double image analysis* (repeatability [76,77])	7/9 patient volumes were suitable for analysis. Accuracy: sagittal 0.7°, 0.4 mm; coronal 0.4˚, 0.2 mm; transverse 0.2°, 0.5 mm Repeatability analysis: 95% of the values were < 2 SD of the mean
Skeppholm 2015 [35]	3D Volume fusion	Patient cervical disc	Subject variability Image analysis	*Double image analysis* (2 observers), ICC, SEM and repeatability	Rotation: ICC 0.95–0.99; SEM 0.34–0.47˚; repeatability 0.93–1.30° Translation: ICC 0.30–0.84; SEM 0.29–0.64 mm; repeatability 0.77–1.78 mm
Svedmark 2015 [36]	3D Volume fusion	Patient lumbar disc	Image analysis	Mean landmark distance after registration (registration error) No reference for 3D movements, comparison pre- post-surgery (*pilot*)	Registration error 0.73 mm No significant difference in rotation or translation between pre- and post-surgery 3D facet joint movement of 3.2–3.6 mm
Wrist					
Reiser 2025 [38]	IMA	Patient carpal and radial comp	Subject variability	Radiography evaluation, no loosening confirmed (*pilot*)	1/3 patients signs of loosening on radiography; 3/3 movement carpal comp between 0.7–3.6 mm and 0.6–7.7° Radial comp no movement detected

CI: 95% confidence interval. Comp: component, CT: computed tomography. ICC: intraclass correlation coefficient, ID-CT: inducible displacement CT, MTPM: maximum total point motion, SD: standard deviation, SEM: standard error of measurement, Sr: repeatability standard deviation, TT: total translation (square root of quadratic sum of x, y, z translations), TR: total rotation (square root of quadratic sum of x, y, z rotations).

#### Diagnostic

The endpoint of ID-CT is to assess primary implant stability or support the diagnosis of aseptic loosening. Accordingly, its performance is evaluated against a reference standard. Across studies, reference standards included intraoperative findings, patient-reported outcomes (PROMs), plain radiographs, inducible displacement RSA, or combinations thereof (see Table 2).

##### Methodological quality

All diagnostic performance studies showed risk of bias in the “Index test” domain due to absence of a prespecified displacement threshold, making results population-specific or unclear (Table S2, see Supplementary data). All studies using intraoperative findings as reference standard were biased because the criteria for loosening were not reported. Additional concerns in the “Flow & timing” domain arose because index test results influenced whether patients underwent revision surgery, which was used as the reference standard (intraoperative findings. These quality limitations—particularly in the choice and application of reference standard(s)—may skew diagnostic accuracy and should be considered when interpreting findings.

##### Outcomes

5 studies assessed ID-CT in cups and stems of THA (see Table 2, hip). At a patient level (either cup or stem loose), sensitivity was 77% [25] and 100% [26], with specificity of 100% in both studies (IMA). Smaller series reported near-perfect classification of loose vs fixed components (7/8 [17], 3D Volume fusion) and 16/20 correct [24], IMA). 1 study compared ID-CT with ID-RSA in stems and found close agreement despite different loading protocols (standing/supine RSA vs internal/external rotation CT) [18] (V3MA).

4 studies evaluated tibial and femoral components of TKA (see Table 2, knee). Using intraoperative findings as reference, sensitivity and specificity were 91% and 72% for tibial [29] (AtMoves), or 100%, and 75% for megaprostheses [33] (IMA). 1 study reported correct identification of all (8) loose components, but fixed components were not confirmed with any reference standard [31] (IMA). Another study in both tibial and femoral components directly compared ID-CT with ID-RSA and found close agreement across most displacement axes, despite differing loading protocols (standing/supine RSA vs standing/sitting CT) [32] (V3MA).

In the spine (see Table 2, spine), 1 study evaluated ID-CT for disc replacement, confirming 3 loose upper components at revision surgery; the remaining 25 patients had no reference standard [35] (3D Volume fusion).

#### Technical

The workflow of ID-CT consists of 3 main steps: load application, CT scanning, and image analysis. Each step may introduce measurement variance, which technical validation studies address by repeating individual steps of the workflow to evaluate 1 or more variance sources—such as subject variability, loading, scanning, or image analysis—depending on the study design (see Table 3). In clinical designs, this was typically done by repeatedly analyzing the same CT scan (pair) by 1 or more observers, i.e., double image analysis, or by acquiring 2 scans of the same patient under the repeated application of the same load, i.e., double examinations [8]. Experimental studies, which were not constrained by radiation exposure, applied additional protocols. These included measuring apparent displacement from repeated scan in unloaded conditions (zero-displacement examinations) and conducting preliminary assessments of ID-CT applicability in specific populations without a reference standard, referred to here as pilot studies.

In hip studies, µCT-analysis in cadavers consistently demonstrated low displacement errors reporting maximal error ranges from 5 to 20 μm across axes [19-22] (see Table 3, hip). Compression-induced displacement reached 5.5 μm [22] to 60 μm [20], while torsion-induced displacement ranged from 49 μm [19] to 119 μm [23], depending on stem design, loading mode, and displacement axis. 2 hip studies evaluated technical validity in clinical settings: 1 used double image analysis to show < 1 mm difference in landmark placement (part of their image analysis pipeline) between 2 observers [17] (IMA), while the other used double examinations and found translation and rotation bias (precision) of 0.055 mm (0.100 mm) and 0.102˚(0.167˚) [18] (V3MA).

In the knee, 2 studies conducted zero-displacement examinations on the same dataset, reporting errors of mean target registration error (mTRE) ≤ 0.08 mm, screw-axis rotation ≤ 0.13˚, and maximum total point motion (MTPM) ≤ 0.12 mm, and clear differentiation between fixed and loose implants, depending on whether a 100% or 20% tibia reference protocol was used (see Table 3, knee) [27,28] (AtMoves). In patients, 1 study repeated the full ID-CT workflow with 2 operators applying the loading device (AtMoves). The study reported varying ICC values (0.17–0.84), depending on the displacement metric and tibia reference protocol, i.e., using either the whole tibia as reference for implant displacement or only the proximal tibia to account for tibial deformation [30]. The same group also assessed double image analysis, reporting good-to-excellent intra- and interrater reliability [29]. Finally, Hext et al. [32] conducted double examinations, finding translation and rotation precision of the tibial and femoral component of 0.056 mm and 0.118˚, vs 0.071 mm and 0.128˚, respectively (V3MA).

In the spine, phantom and patient studies (using 3D Volume fusion) evaluated accuracy, repeatability, and registration error (see Table 3, spine). Phantom disc models showed accuracy of 0.20–0.56 mm depending on displacement axis [34,37]. Skeppholm et al. [35] evaluated double image analysis and reported excellent reliability for rotation (ICC 0.95–0.99), but lower for translation (ICC 0.30–0.84), attributed to small translations in the coronal plane. A pilot lumbar study from the same group reported a registration error of 0.73 mm, which was below the observed facet joint displacement (3.2–3.6 mm) [36].

In the wrist, a pilot study evaluated carpal and radial component in 3 patients (see Table 3, wrist), reporting carpal component displacement in all patients (0.7–3.6 mm, 0.6–7.7˚), while no displacement was detected in the radial component [38] (IMA).  

## Discussion

This scoping review summarized literature on inducible displacement CT (ID-CT) for implant loosening detection, focusing on loading protocols, CT acquisition, image analysis, and validation strategies. Twenty-two studies, covering hip [17-26], knee [27-33], cervical spine [34,35], lumbar spine [36,37] and wrist [38], showed variation in loading protocol and CT acquisition settings. Image analysis workflows were generally consistent but often lacked transparency in processing methods and displacement metrics. Validation strategies also differed in both diagnostic reference standards and technical methods. Reported diagnostic performance—although high—should be interpreted in the light of the identified risk of bias across the index test, reference standard, and flow & timing domains.

CT acquisition settings and effective dose (ED) in ID-CT are similar to those used in CT migration analysis protocols, where minimizing radiation exposure is critical given the 2–8 scans often required per patient [39]. While ID-CT typically requires only 2 scans, the use of low-dose protocols (ED < 1 mSv) remains important, especially in high-conversion regions such as the hip, spine, and shoulder. Recent CT migration analysis studies report EDs of 0.02–0.09 mSv (knee) [40-44], 0.27–1.54 mSv (shoulder) [2,45,46], and often ≥ 1 mSv in the hip [47-53]. Øhrn et al. [54] found that dose reduction does not compromise precision, though detailed acquisition and reconstruction settings were not reported. A key limitation of dose reduction is the potential increase in metal artefacts. In both CT migration analysis and ID-CT, this is mitigated through the use of metal artefact reduction (MAR) reconstruction algorithms [2,18,25,33,38,40,41,52,55,56]. While displacement analysis is generally robust, the effect of MAR reconstruction on segmentation and registration accuracy remains uncertain [57].

ID-CT and CT migration analysis rely on shared methods and evidence. Both use “double examinations” to evaluate bias and random error, either by repeating scans of the same patient on the same day (CT migration analysis) [8] or under the same loading condition (ID-CT). Van de Vusse et al. [10] referred to the standard deviation from such examinations as “clinical precision,” reporting ranges from 0.03–1.36 mm and 0.06–2.25°. 2 of the 8 studies they reviewed applied ID-CT [18,34] and were included in this scoping review. A third study reported clinical precision values of 0.06 mm and 0.12° for the tibial, and 0.07 mm and 0.13° for the femoral component [32]. Although this review identified several robust attempts to validate ID-CT—addressing variance across subjects, CT acquisition, and image analysis—most study designs do not assess how these errors accumulate across the entire workflow, with the role of the loading protocol underemphasized and assessed in only 2 studies [19,30].

The lack of standardized loading protocols and inconsistent reporting of compliance hinder comparability across studies and may contribute to variability in measurements. Manual loading depends on the operator, patient, and session, and was often guided by subjective endpoints such as pain or range of motion. This variability is evident across anatomical regions: hip studies typically used manual torsional loading with inconsistent stabilization [17,18,24-26], while spine studies relied on subjective flexion–extension positioning [34-36], both approaches compromising reproducibility. For the knee, dedicated mechanical loading devices have been developed, with Buijs et al. [30] reporting moderate-to-good inter-operator reliability using a standardized 4-point bending loading device with repeated cycles. Weightbearing methods, such as those studied by Hext et al. [32], remain influenced by posture, muscle activation, and compliance.

Without harmonized, well-documented protocols, site- and operator-dependent differences continue to limit clinical generalizability and the establishment of uniform diagnostic thresholds. Across the included studies, loosening criteria were either retrospectively determined or not (clearly) specified, and displacement metrics varied considerably, with most studies relying on qualitative or semi-quantitative assessment. Listopadzki et al. [26], for example, reported a loosening threshold of > 0.5 mm while assessing both translation and rotation [58], without a clear rationale for this cut-off. Buijs et al. [59] retrospectively derived quantitative loosening thresholds using multiple metrics (0.53˚ screw axis rotation, 0.42 mm mTRE, and 0.70 mm MTPM), with MTPM being most comparable to RSA-based migration measures [60]. In RSA, MTPM thresholds have been linked to revision risk, with 6-month mean MTPM considered acceptable below 0.30 or 1.10 mm and unacceptable above 1.10 or 1.55 mm for cemented and uncemented tibial components, respectively [1], underscoring the potential value of quantitative criteria for ID-CT. A pragmatic starting point would be that displacement exceeding the measurement error may indicate loosening; however, relying solely on this risks overestimating loosening, as apparent displacement may partly reflect elastic deformation of the surrounding bone rather than true implant loosening [28]. At present, ID-CT loosening thresholds should be interpreted cautiously, with future work focusing on standardized quantitative criteria that account for measurement error and elastic bone deformation.

Another challenge in ID-CT is the lack of a uniform clinical reference standard for validating implant loosening. Most studies use revision surgery findings, supplemented by radiography, PROMs, or comparisons with ID-RSA. Although the only ethical choice, verifying only patients with suspected loosening inflates sensitivity and leaves specificity undefined. Standardized intraoperative definitions of implant stability (e.g., visible fluid motion, as recently defined by Delphi consensus for TKA [61]) and structured follow-up for non-revised patients are needed. Follow-up should integrate PROMs and radiography, recognizing that radiolucent lines alone have poor specificity but gain diagnostic value when combined with symptoms [62-68]. Additionally, many studies lacked surgeon blinding to ID-CT results [25,26,31,33,35], introducing potential observer bias and underscoring the need for blinded, multicenter validation trials.

### Limitations

A limitation of this scoping review is that study selection, data extraction, and critical appraisal were conducted by a single reviewer, which may introduce bias. However, the use of predefined inclusion criteria, a structured data extraction table, and explicit reporting of reasons for identified risk of bias helped mitigate that risk.

In perspective, the challenges highlighted in this review translate into practical considerations for future ID-CT studies, as outlined in the Supplementary data (S3). Because no study met all QUADAS-2 quality criteria, the diagnostic reliability is still not fully established, though transparently reported techniques such as AtMoves [27-30] and V3MA [18,32] currently offer valuable methodological insights. True diagnostic accuracy, however, requires blinded multicenter trials. The premature clinical application of ID-CT carries risks, including unnecessary radiation exposure and misdiagnosis. This results not from validated diagnostic accuracy, but from the perceived sophistication and novelty of the technique.

### Conclusion

This scoping review demonstrates that inducible displacement CT studies are performed using highly heterogeneous loading protocols, CT settings and dose, and validation strategies. Although most studies follow a broadly similar image analysis workflow, methodological differences and incomplete reporting limit reproducibility and comparability across studies. Moreover, the current literature does not allow determination of true diagnostic accuracy, as diagnostic studies showed risk of bias related to reference standards, non-prespecified thresholds, and lack of blinding. A key methodological challenge remains in differentiation of true implant displacement from measurement error, which may be introduced at any step of the ID-CT pipeline. Validation covering the entire pipeline, together with transparent and standardized reporting, is therefore essential to identify sources of variance and support methodological optimization. The practical considerations outlined in this review provide an initial framework toward standardization, which is a prerequisite for site-independent diagnostic thresholds and enablement of broader clinical adoption of ID-CT for the assessment of implant loosening.

### Supplementary data

ID-CT characteristics Table (S1), QUADAS-2 Diagnostic studies (S2), ID-CT Practical considerations (S3), and references are available as Supplementary data on the article home page, doi: 10.2340/17453674.2026.45512

## Supplementary Material



## Notes

The project was initiated by MW and coordinated by GS and ID. MW performed study selection and data extraction, and wrote the initial draft. All members (ID, AK, MS, MM, LB, GS) revised the manuscript.

Handling co-editor: Per Henrik Randsborg

*Acta* thanks Stephan Maximilian Röhrl and other anonymous reviewers for help with peer review of this manuscript.
