# Supplementary material for: Inducible displacement CT for implant loosening detection: a scoping review on methods, validation, and challenges
Source: Acta Orthop. 2026 Feb 23;97:136–47. doi: 10.2340/17453674.2026.45512 (PMC12927442; doi:10.2340/17453674.2026.45512)
Supplement: Supplementary file 1 [file ActaO-97-45512-s1.pdf]

Supplemental material

Contents

1. ID-CT characteristics table (S1)
2. QUADAS-2 Diagnostic studies (S2)
3. ID-CT Practical considerations (S3)
4. References

Table S1

Characteristics of ID-CT studies included in this review, grouped by anatomical site. Validation attempts of ID-CT were broadly divided into *diagnostic*, evaluating the diagnostic performance (Table 2), or *technical*, assessing accuracy, precision (i.e., repeatability) and robustness of ID-CT (Table 3).

	Study	Component(s)	Design	Validation	Sample size	Loading protocol	CT parameters	Image analysis	Displacement metrics
Hip	Olivecrona 2008 [1]	THA – Acetabular cup	Prospective clinical	Diagnostic & Technical	8/10 patients	Torsional loading (manual held by examiner)	GE LightSpeed. 120 kV, 200 mA, ST=1.25 mm, PS=1.30-1.66 mm, ED=3.5mSv per scan[1]	3D volume fusion tool (landmark-based rigid body transformation) [2-4]	Visual assessment of motion.
	Gortchacow 2011 [5]	THA- femoral stem	Cadaveric experimental	Technical	1 femoral bone	Compression. Single cycle stepwise loading (0, 300, 600, 900, 1200, 1400, 0N) with custom loading device.	Micro-CT (Skyscan 1076). 100kV, 24 mAs[1]. VS=36 µm (isotropic)	5 Ta beads in stem, 15 Ta beads in medullary bone. Custom automatic (gray-scale based) bead centre of mass detection. Image processing and analysis in Mathematica (Wolfram Research)	Local CS placed in unloaded stem; x-axis ML, y-axis AP, z-axis IS. X, Y, Z displacement.
	Gortchacow 2012 [6]	THA – femoral stem (anatomical and straight)	Cadaveric experimental	Technical	6 femoral bones (3 cadavers)	Compression (2000N). Single cycle loading sequence (unloaded, loaded, unloaded, unloaded) with custom loading device.	Micro-CT (Skyscan 1076). VS=36 µm (isotropic)	Custom algorithm for bead centre of mass detection [5].	Local CS with displacements similar to [5]
	Malfroy Camine 2015 [7]	THA – femoral stem	Cadaveric experimental	Technical	1 femoral bone	Compression (1800N). Single cycle loading sequence (unloaded, loaded, unloaded, loaded, unloaded) with custom loading device.	Micro-CT (Skyscan 1076). VS=35 µm (isotropic)	Beads identification and location in Amira (FEI, USA).<br><br>30 Ta beads in stem, 1000 SS beads on endosteal surface.	Unloaded (4th) scan used as reference for Local CS with displacements similar to [5]
	Malfroy Camine 2016 [8]	THA – femoral stem	Cadaveric experimental	Technical	1 femoral bone	Compression (1800N) + Torsion (17Nm). Triple cycle (loaded, unloaded) in custom loading devices (compression device modified from [6])	Micro-CT (Skyscan 1076). 100 kV, ~32 mAs[2], VS=35 µm (isotropic). Ring artefact and beam-hardening correction (NRecon v 1.6.10.4, Bruker)	Custom Matlab algorithm + Amira (v6.0.1 FEI) segmentation. 30 Ta beads in stem, ~1000 beads on endosteal surface.	Unloaded scan used as reference for Local CS. Displacements (normal and tangential to stem surface) similar to x,y or z-axis in [5]
	Malfroy Camine 2018 [9]	THA – femoral stem (4 collarless and 6 collared)	Cadaveric experimental	Technical	10 femoral bones (10 cadavers)	Compression (load corresponding to 230% donor's body BW) + Torsion (moment corresponding to 2.3% BW x m). Single cycle (unloaded, loaded, unloaded) in loading devices similar to [8]	Similar to [8]	Custom Matlab algorithm (unspecified) 37 Ta beads in stem, ~1000 beads on endosteal surface.	Unloaded (3rd) scan used as reference for Local CS. Displacements normal and tangential to stem surface, similar to [8] and 3D (absolute) vector.
	Sandberg 2021 [10]	THA – both components + 1 knee tumor prosthesis	Retrospective clinical	Diagnostic	10/60 patients	Torsional loading (external stabilization with sandbags)	unspecified	(unspecified) local developed software, IMA	Qualitative assessment: positive/negative for loosening
	Sandberg 2022 [11]	THA – both components	Retrospective clinical	Diagnostic	57/72 patients	Torsional loading (external stabilization with sandbags). Pain-limited	Siemens Somatom. 120kV, 180mAs, ST=0.6 mm. with/without iMAR. ED=4.50mSv	IMA (Sectra), methods developed by [2, 12]	Qualitative: positive/negative for movement. Quantitative: "mm and deg" when movement detected (threshold or metric unspecified)
	Listopadzki 2025 [13]	THA – both components	Retrospective clinical	Diagnostic	23/80 patients	Torsional loading (with external stabilization with foam pads and weighted bags)	130kV, 100-130 mAs, AD=13-14mGy per scan	IMA (Sectra) [2, 12]	Implant motion >0.5mm threshold. Qualitative: micromotion and "spotwelding" assessment at implant-bone/cement interface
	Polus 2025 [14]	THA – femoral stem	Prospective clinical	Diagnostic & Technical	48/79 patients	Torsional loading (manual)	Aquilion ONE, Canon. 120 kVp, ST=0.5mm, PS= 0.45± 0.04 mm, ED=1.51 ± 0.80 mSv per scan. SEMAR.	Segmentation in 3DSlicer, registration in V3MA	X,Y, Z and total translation/rotation (TT/TR). RSA CS (x-axis ML, y-axis IS, z-axis AP)
Knee	Wretenberg 2021 [15]	TKA – both components	Clinical series	Diagnostic	40 patients	Torsional and varus-valgus loading (with external fixation with lashing straps, foam pads and weighted bags)	Not specified	IMA (Sectra)	Visual assessment

	Study	Component(s)	Design	Validation	Sample size	Loading protocol	CT parameters	Image analysis	Displacement metrics
	Kievit & Buijs 2023 [16]	TKA – tibial component	Cadaveric experimental	Technical	10 specimens + 1 frozen specimen	Varus-valgus loading device (20 Nm)	Brilliance-64 Philips. VS=0.3mm (isotropic)	Custom 3D analysis software [17]	mTRE, screw-axis rotation, MTPM.
	Ter Wee 2023 [18]	TKA – tibial component	Cadaveric experimental	Technical	10 specimens + 1 frozen specimen from [16]	Varus-valgus loading device (20 Nm)	Brilliance-64 Philips. 120 kV, 160 mAs, VS=0.45mm (isotropic)	Custom 3D analysis software [19]	Comparison of 20% vs 100% tibia reference for quantifying implant displacement. Metrics similar to [16]
	Buijs & Kievit 2025 [20]	TKA – tibial component	Prospective clinical	Diagnostic & Technical	34 symptomatic + 38 asymptomatic knees	Varus-valgus loading device (20 Nm)	Estimate ED=1.2mSv	Custom 3D analysis software [17]	mTRE, screw-axis rotation, MTPM
	Buijs & Ter Wee 2025 [21]	TKA – tibial component	Inter-operator reliability study	Technical	16 patients	Varus-valgus loading (20 Nm, 2 operators)	Siemens Somatom Force, 120 kVp, 160 mAs. VS = 0.45mm (isotropic)	AtMoves system	mTRE, screw-axis rotation, MTPM
	Hext 2025 [22]	TKA – both components	Prospective clinical	Diagnostic & Technical	17 patients	Weightbearing (seated vs. standing)	OnSight 3D Extremity CT System. 90 kVp, 20—45 mAs, ST=0.26mm, PS=0.26mm. ED=0.04-0.06mSv.	Segmentation in 3DSlicer, registration in V3MA	X,Y, Z and total translation/rotation (TT/TR), and MTPM.<br>RSA CS (x-axis ML, y-axis IS, z-axis AP)
	Svensson 2025 [23]	TKA – megaprostheses	Retrospective clinical	Diagnostic	9/12 patients (13 exams)	Torsional lading (external and internal rotation limit)	Reference to [11]	IMA (Sectra)	Visual and quantitative assessment
Spine	Svedmark 2008 [24]	Lumbar disc replacement	Phantom model experimental	Technical	104 CT volumes	Controlled mechanical translation	LightSpeed QX/i fourth-generation GE. 120 kV, 250 mA.	3D volume fusion tool (landmark-based rigid body transformation) [25]	3D sphere landmark translation. DICOM CS
	Svedmark 2011 [26]	Cervical disc replacement - Upper and lower component.	Prospective clinical + phantom model experimental	Technical	9 patients + cervical spine model	Voluntary flexion-extension positioning, with head on soft/stiff pillow	Patients: Siemens Somatom Definition. 140 kVp, 47 mAs. PS=0.22-0.29mm. ED=0.33 mSv<br><br>Model: GE Lightspeed. 120 kVp, 1.722 mAs. PS=0.39mm.	3D volume fusion tool (landmark-based rigid body transformation) [2, 25, 27]	Euler angles (sequence RzRyRx) and orthogonal translation (x,y,z)<br><br>Patients: Visual assessment<br><br>Local CS based on upper components and aligned with DICOM CS
	Skeppholm 2015 [28]	Cervical disc replacement – Upper and lower component.	Prospective clinical	Diagnostic & Technical	28 patients (38 levels)	Voluntary flexion-extension positioning, with head on stiff pillow	Siemens Somatom Definition. ED=0.33 mSv.	3D volume fusion tool (landmark-based rigid body transformation). Similar to [26].	Rotation and translation in all planes<br><br>Any detectable motion between prosthetic components is 'ankylotic'. Any detectable motion between components and vertebrae is 'loose'.
	Svedmark 2015 [29]	Lumbar disc replacement	Prospective clinical	Technical	10 patients	Provoked flexion (prone) and extension (supine) using a customized jig. Provocation till VAS 8/10 or top of CT scanner tunnel	GE Light Speed QX/I. 120 kV, 250 mA. PS=4mm. ED=0.68mSv	3D volume fusion tool (landmark-based rigid body transformation). Similar to [26].	Segmental 3D rotation (Euler angles sequence RzRyRx) and 3D translation between both L4-L5 and L5-S1. Evaluation of ROM.<br><br>DICOM CS
Wrist	Reiser 2025 [30]	TWA - carpal and radial component	Pilot clinical	Technical	3 patients	Flexion-extension with orthoses	Siemens Somatom Definition. 120 kV, 100 mAs, ST=0.6mm. with/without iMAR. 0.01 mSv ED	IMA (Sectra)	Movement assessment of carpal component vs. third metacarpal and capitate, and radial component vs. radius.

Abbreviations per column: **Component(s)**: THA; Total hip arthroplasty. TWA; Total Wrist Arthroplasty. **Loading protocol:** VAS; Visual Analog Score [31], BW; Body Weight. **CT parameters:** ST; Slice Thickness, PS; Pixel Size, VS; Voxel Size, ED; Effective Dose, AD; Absorbed Dose, iMAR; iterative metal artefact reduction (Siemens), SEMAR; single energy metal artifact reduction (Canon Medical System Corporation). **Image analysis:** Ta; Tantalum, SS; stainless steel, IMA; Implant Motion Analysis (Sectra, Linköping, Sweden), V3MA; Volumetric Matching Micromotion Analysis (RSAcore, Leiden, The Netherlands). **Displacement metrics:** CS; Coordinate System. ML; medio-lateral. AP; antero-posterior. IS; inferior-superior, TT/TR; Total Translation/Total Rotation, MTPM; Maximum Total Point Motion, DICOM; Digital Imaging and Communications in Medicine, RSA; Radiostereometric analysis. *Footnotes*: 1. By using only segments of the scans around the hip, ED was calculated to be 2.5 mSv. 2. Reported tube current (100 µA) and exposure time (240 ms). 2. Reported is the tube current (100 µA), exposure time per frame (310 ms), Rotation step (0.7˚), Frame averaging (2) which can be calculated to the mAs.

Table S2 Risk of bias assessment (QUADAS-2)

Risk of bias and applicability assessment of included diagnostic studies using the QUADAS-2 tool. Reasons for negative responses are provided below the table.

		**Risk of bias**				**Applicability**		
		Patient selection	Index test	Reference standard	Flow & timing	Patient selection	Index test	Reference standard
Hip	Olivecrona 2008 [1]	✓	✗[1b]	✗[2]	✓	✓	✓	✓
	Sandberg 2021 [10]	✓	✗[1b]	✗[2]	✓	✓	✓	✓
	Sandberg 2022 [11]	✓	✗[1b]	✗[2,3]	✗[4]	✓	✓	✓
	Listopadzki 2025 [13]	✓	✗[1b]	✗[2,3]	✗[4]	✓	✓	✓
	Polus 2025 [14]	✓	✗[1a]	✓	✓	✗[5]	✓	✓
Knee	Wretenberg 2021 [15]	✓	✗[1b]	✗[2,3]	✗[4]	✓	✓	✓
	Buijs & Kievit 2025 [20]	✓	✗[1a]	✗[2]	✓	✓	✓	✓
	Hext 2025 [22]	✓	✗[1a]	✓	✓	✗[5]	✓	✓
	Svensson 2025 [23]	✓	✗[1b]	✗[2,3]	✗[4]	✓	✓	✓
Spine	Skepholm 2015 [28]	✓	✗[1b]	✗[2,3]	✗[4]	✓	✓	✓

Reasons for risk of bias: 1) The diagnostic threshold was (a) retrospectively determined and/or (b) not/unclearly defined. 2) Intraoperative findings used as the reference standard lacked details or methodology for classifying loosening. 3) Results of the reference standard were interpreted with knowledge of the results of the index text. 4) Patients received different reference standards. **Reasons for concerns about applicability:** 1) Only patients with fixed components (per RSA), with no suspicion of loosening.

S3: ID-CT Practical considerations

Current studies on ID-CT carry a high risk of bias, preventing confident determination of its diagnostic accuracy (Table S2). Techniques that report their methods transparently, such as AtMoves, V3MA, and µCT-analysis, demonstrate the potential of ID-CT and offer a roadmap for reproducible technical and diagnostic evaluation. Ensuring such transparency is critical for fair comparison across studies to guide future clinical implementation based on robust evidence rather than the perceived novelty of the technique.

Standardization and reporting guidelines diagnostic studies

The recently updated guidelines for RSA and CT-RSA (i.e. CT migration analysis) implant migration measurements [32] provides foundation that can be adapted for ID-CT studies. However, ID-CT differs in that it aims at immediate diagnosis of loosening rather than long-term monitoring of implant survival. Hence, shifting to evaluating diagnostic accuracy and therefore necessitating adherence to the Standards for Reporting of Diagnostic Accuracy Studies (STARD 2015 [33]).
In addition to CT-RSA guidelines, essential reporting items for ID-CT studies should include the loading protocol and reference standard. At minimum, studies should report:

Loading protocol specifications:

- Description of loading device or manual technique, including clear visualization of the setup
- Metric (quantitative or qualitative) that determines loading endpoint, for example moment readout [21] or pain score [29]. The use of subjective endpoints should be, where possible, combined with an objective measurement of joint position on CT table or from the CT scan.
- Patient compliance metrics, including target load achieved, duration, and deviation from the prescribed load.
- Patient positioning on CT table in both loading conditions, including joint orientation angles (for example knee flexion angle).
- Reference citations to prior technical validation studies.
- For experimental studies, detailed reporting of specimen preparation and loading sequences.

Reference standard documentation:

- Clear definition of loose versus fixed classification criteria, following STARD 2015 item 12b [33].
- For knee arthroplasty, the consensus definition by Buijs et al. [34] provides standardized intraoperative criteria; comparable standardized definitions are needed for hip, spine, and wrist applications.

Technical validation considerations

Technical validation of ID-CT methods requires assessment of variance introduced at each step of the diagnostic pipeline: loading application, CT acquisition, and image analysis. While 'double examinations' following the RSA standard ISO 16087:2013 [35] can evaluate CT acquisition and image analysis reproducibility, they do not capture the full diagnostic pipeline including loading variations. Underestimating for example the contribution of bone deformation in measurements of tibial component displacement [18]. Complete assessment of ID-CT reproducibility should include both loading conditions, as demonstrated by Malfroy Camine et al. [8] in a cadaveric study and Buijs & Ter Wee et al. [21] in a clinical setting.

References

1. Olivecrona, H., et al., *A new technique for diagnosis of acetabular cup loosening using computed tomography: preliminary experience in 10 patients.* Acta Orthop, 2008. **79**(3): p. 346–53.
2. Olivecrona, L., et al., *Acetabular component migration in total hip arthroplasty using CT and a semiautomated program for volume merging.* Acta Radiol, 2002. **43**(5): p. 517–27.
3. Olivecrona, H., et al., *Stability of acetabular axis after total hip arthroplasty, repeatability using CT and a semiautomated program for volume fusion.* Acta Radiol, 2003. **44**(6): p. 653–61.
4. Olivecrona, L., et al., *Model studies on acetabular component migration in total hip arthroplasty using CT and a semiautomated program for volume merging.* Acta Radiol, 2003. **44**(4): p. 419–29.
5. Gortchacow, M., et al., *A new technique to measure micromotion distribution around a cementless femoral stem.* Journal of Biomechanics, 2011. **44**(3): p. 557–560.
6. Gortchacow, M., et al., *Simultaneous and multisite measure of micromotion, subsidence and gap to evaluate femoral stem stability.* Journal of Biomechanics, 2012. **45**(7): p. 1232–1238.
7. Malfroy Camine, V., et al., *Distribution of gap and micromotion during compressive loading around a cementless femoral stem.* Comput Methods Biomech Biomed Engin, 2015. **18 Suppl 1**: p. 1896–7.
8. Malfroy Camine, V., et al., *Full-field measurement of micromotion around a cementless femoral stem using micro-CT imaging and radiopaque markers.* J Biomech, 2016. **49**(16): p. 4002–4008.
9. Malfroy Camine, V., et al., *Effect of a collar on subsidence and local micromotion of cementless femoral stems: in vitro comparative study based on micro-computerised tomography.* Int Orthop, 2018. **42**(1): p. 49–57.
10. Sandberg, O., H. Olivecrona, and P. Gustafson, *Adverse Events due to Lack of Precision in Total Hip Arthroplasty: The Potential of Provocation-Based CT for Diagnosis of Implant Loosening.* ScientificWorldJournal, 2021. **2021**: p. 8836687.
11. Sandberg, O., et al., *Inducible displacement CT increases the diagnostic accuracy of aseptic loosening in primary total hip arthroplasty.* Acta Orthop, 2022. **93**: p. 831–836.
12. Jedenmalm, A., et al., *A new approach for assessment of wear in metal-backed acetabular cups using computed tomography: a phantom study with retrievals.* Acta Orthop, 2008. **79**(2): p. 218–24.
13. Listopadzki, T.R., K.K. Boyle, and S.R. Nodzo, *Computed Tomography With Implant Movement Analysis in the Work-Up of Painful Total Hip Prostheses.* J Arthroplasty, 2025.
14. Polus, J.S., et al., *Evaluation of conventional and CT-based radiostereometric analysis for inducible displacement measurements after total hip arthroplasty.* J Orthop Res, 2025. **43**(1): p. 192–199.
15. Wretenberg, P., et al., *Implant Movement Analysis (IMA), A New CT Based Technique for Diagnosis of Aseptic Loosening of Total Knee Arthroplasty.* Orthopedic Research Online Journal, 2021. **8**(3).
16. Kievit, A.J., et al., *Promising results of an non-invasive measurement of knee implant loosening using a loading device, CT-scans and 3D image analysis.* Clin Biomech (Bristol), 2023. **104**: p. 105930.
17. Dobbe, J.G.G., et al., *Evaluation of a CT-based technique to measure the transfer accuracy of a virtually planned osteotomy.* Medical Engineering & Physics, 2014. **36**(8): p. 1081–1087.
18. Ter Wee, M.A., et al., *Load-induced deformation of the tibia and its effect on implant loosening detection.* Sci Rep, 2023. **13**(1): p. 21769.

19. Dobbe, J.G.G., et al., *Evaluation of a Quantitative Method for Carpal Motion Analysis Using Clinical 3-D and 4-D CT Protocols.* IEEE Transactions on Medical Imaging, 2019. **38**(4): p. 1048–1057.
20. Buijs, G.S., et al., *Non-invasive quantitative assessment of induced component displacement can safely and accurately diagnose tibial component loosening in patients: A prospective diagnostic study.* Knee Surg Sports Traumatol Arthrosc, 2025. **33**(1): p. 274–285.
21. Buijs, G.S., et al., *Operator variation in applying a knee loading device for evaluation of tibial component loosening in total knee arthroplasty.* Clin Biomech (Bristol), 2025. **126**: p. 106531.
22. Hext, R.A., et al., *Inducible displacement of cementless total knee arthroplasty components with conventional and weight-bearing CT-based radiostereometric analysis.* J Orthop Res, 2025. **43**(3): p. 640–649.
23. Svensson, P.A., P. Tsagkozis, and H. Olivecrona, *CT-based Implant Motion Analysis for diagnosing loosening of megaprostheses : validation in a clinical setting.* Bone Joint J, 2025. **107-b**(5): p. 556–560.
24. Svedmark, P., et al., *Model studies on segmental movement in lumbar spine using a semi-automated program for volume fusion.* Comput Aided Surg, 2008. **13**(1): p. 14–22.
25. Noz, M.E., et al., *A versatile functional-anatomic image fusion method for volume data sets.* J Med Syst, 2001. **25**(5): p. 297–307.
26. Svedmark, P., et al., *Motion analysis of total cervical disc replacements using computed tomography: preliminary experience with nine patients and a model.* Acta Radiol, 2011. **52**(10): p. 1128–37.
27. Gorniak, R.J., et al., *Evaluation of a semiautomatic 3D fusion technique applied to molecular imaging and MRI brain/frame volume data sets.* J Med Syst, 2003. **27**(2): p. 141–56.
28. Skeppholm, M., et al., *Evaluation of mobility and stability in the Discover artificial disc: an in vivo motion study using high-accuracy 3D CT data.* J Neurosurg Spine, 2015. **23**(3): p. 383–9.
29. Svedmark, P., et al., *A New CT Method for Assessing 3D Movements in Lumbar Facet Joints and Vertebrae in Patients before and after TDR.* Biomed Res Int, 2015. **2015**: p. 260703.
30. Reiser, D., et al., *CT Motion-Analysis of Implant Loosening in Total Wrist Arthroplasty: A Pilot Study.* Journal of Wrist Surgery, 2025.
31. McCormack, H.M., D.J. de L. Horne, and S. Sheather, *Clinical applications of visual analogue scales: a critical review.* Psychological Medicine, 1988. **18**(4): p. 1007–1019.
32. Kaptein, B.L., et al., *Guideline for RSA and CT-RSA implant migration measurements: an update of standardizations and recommendations.* Acta Orthop, 2024. **95**: p. 256–267.
33. Cohen, J.F., et al., *STARD 2015 guidelines for reporting diagnostic accuracy studies: explanation and elaboration.* BMJ Open, 2016. **6**(11): p. e012799.
34. Buijs, G.S., et al., *Visible fluid motion on manipulation as the new threshold for intraoperatively determined knee arthroplasty component loosening: A Delphi study.* Knee Surgery, Sports Traumatology, Arthroscopy, 2025. **33**(1): p. 343–353.
35. Standardization, I.O.f., *Implants for surgery — Roentgen stereophotogrammetric analysis (RSA) of implant movement*. 2013: Geneva, Switzerland.
